# Identification and Validation of Cuproptosis-Related LncRNA Signatures in the Prognosis and Immunotherapy of Clear Cell Renal Cell Carcinoma Using Machine Learning

**DOI:** 10.3390/biom12121890

**Published:** 2022-12-16

**Authors:** Zhixun Bai, Jing Lu, Anjian Chen, Xiang Zheng, Mingsong Wu, Zhouke Tan, Jian Xie

**Affiliations:** 1 Organ Transplant Center, The Affiliated Hospital of Zunyi Medical University, Zunyi 563006, China; 2 Department of Nephrology, The Affiliated Hospital of Zunyi Medical University, Zunyi 563006, China; 3 Department of Clinical, Zunyi Medical and Pharmaceutical College, Zunyi 563000, China; 4 Department of Urology, The Affiliated Hospital of Zunyi Medical University, Zunyi 563006, China; 5 Department of Medical Genetics, Zunyi Medical University, Zunyi 563006, China

**Keywords:** ccRCC, lncRNA, cuproptosis, immunotherapy, TMB score

## Abstract

(1) Objective: We aimed to mine cuproptosis-related LncRNAs with prognostic value and construct a corresponding prognostic model using machine learning. External validation of the model was performed in the ICGC database and in multiple renal cancer cell lines via qPCR. **(2)** Methods: TCGA and ICGC cohorts related to renal clear cell carcinoma were included. GO and KEGG analyses were conducted to determine the biological significance of differentially expressed cuproptosis-related LncRNAs (CRLRs). Machine learning (LASSO), Kaplan–Meier, and Cox analyses were conducted to determine the prognostic genes. The tumor microenvironment and tumor mutation load were further studied. TIDE and IC50 were used to evaluate the response to immunotherapy, a risk model of LncRNAs related to the cuproptosis genes was established, and the ability of this model was verified in an external independent ICGC cohort. LncRNAs were identified in normal HK-2 cells and verified in four renal cell lines via qPCR. (3) Results: We obtained 280 CRLRs and identified 66 LncRNAs included in the TCGA-KIRC cohort. Then, three hub LncRNAs (AC026401.3, FOXD2−AS1, and LASTR), which were over-expressed in the four ccRCC cell lines compared with the human renal cortex proximal tubule epithelial cell line HK-2, were identified. In the ICGC database, the expression of FOXD2-AS1 and LASTR was consistent with the qPCR and TCGA-KIRC. The results also indicated that patients with low-risk ccRCC—stratified by tumor-node metastasis stage, sex, and tumor grade—had significantly better overall survival than those with high-risk ccRCC. The predictive algorithm showed that, according to the three CRLR models, the low-risk group was more sensitive to nine target drugs (A.443654, A.770041, ABT.888, AG.014699, AMG.706, ATRA, AP.24534, axitinib, and AZ628), based on the estimated half-maximal inhibitory concentrations. In contrast, the high-risk group was more sensitive to ABT.263 and AKT inhibitors VIII and AS601245. Using the CRLR models, the correlation between the tumor immune microenvironment and cancer immunotherapy response revealed that high-risk patients are more likely to respond to immunotherapy than low-risk patients. In terms of immune marker levels, there were significant differences between the high- and low-risk groups. A high TMB score in the high-risk CRLR group was associated with worse survival, which could be a prognostic factor for KIRC. (4) Conclusions: This study elucidates the core cuproptosis-related LncRNAs, FOXD2−AS1, AC026401.3, and LASTR, in terms of potential predictive value, immunotherapeutic strategy, and outcome of ccRCC.

## 1. Introduction

Renal cell carcinoma (RCC) is a malignant tumor originating from the renal tubular epithelium, accounting for 80–90% of malignant renal tumors. Annually, approximately 430,000 new RCC cases are diagnosed and 180,000 deaths occur worldwide [1]. Clear cell renal cell carcinoma (ccRCC) is the most common type of RCC, accounting for approximately 60% of all cases and 75–80% of metastatic cases [2]. Despite advances in surgery, radiotherapy, and chemotherapy, which have significantly improved over the past decades, there has been no significant change in the five-year survival rate for ccRCC, and it remains a highly aggressive cancer with recurrence. Advanced renal cancer, in particular, is not sensitive to chemotherapy drugs; thus, relevant medical treatment mainly includes immunotherapy and targeted therapy. Compared with targeted therapy, immunotherapy has a slow onset and low effective rate, but its effects are long-lasting. Therefore, the best treatment model for advanced renal cancer may be immunotherapy combined with targeted therapy, which can rapidly reduce tumor load while allowing patients to survive for a long time.

Cell death induction, including apoptosis, necroptosis, pyroptosis, ferroptosis, and autophagy, is always the core mechanism of antitumor drugs. Scientists have recently discovered a new type of cell death—namely, cuproptosis [3]. This occurs through the direct binding of copper ions to lipoacylated components of the tricarboxylic acid cycle in mitochondrial respiration, resulting in the aggregation of lipoacylated proteins and subsequent down-regulation of iron–sulfur cluster proteins, as well as protein-toxic stress, and ultimately, cell death. Thus, cuproptosis may serve as a possible combination treatment for cancer [4].

A long non-coding RNA (lncRNA) has a length greater than 200 nucleotides but is unable to code for proteins. Abnormal lncRNA expression plays an important role in ccRCC occurrence, development, metastasis, and prognosis [5,6]. This provides a new direction for the study of the molecular mechanisms, diagnosis, and treatment of RCC [7]. When a new lncRNA meets the new method of cell death—that is, cuproptosis—and the associated new immunotherapy, it is reasonable to speculate that the new combination will inevitably lead to the emergence of new treatment and prevention strategies for ccRCC.

Machine learning (ML), a branch of artificial intelligence, is widely defined as a group of computer-aided strategies. ML automatically determines the methods and parameters needed to obtain the best solution to a given research problem [8]. ML classifiers currently provide new research methods for interdisciplinary big data research [9,10]. For example, ML methods have been used to try to improve the accuracy of clinical research predictions [11,12].

In this study, we mined the ccRCC database in TCGA to analyze the correlation between cuproptosis-related genes, LncRNAs, and the tumor microenvironment using machine learning and bioinformatic analyses. Subsequently, 12 potential target drugs—inhibitors of Akt, BCL2, PARP, VEGFR, TKI, JNK, and Raf—were selected as being correlated with the cuproptosis-related LncRNAs (CRLRs) and immune microenvironment. In this way, we reveal the potential prognostic value, immunotherapeutic strategy, and ccRCC outcomes.

## 2. Materials and Methods

### 2.1. ccRCC Data Collection

ccRCC patient data (72 normal patients and 539 ccRCC patients) were downloaded from the TCGA database. We collected clinical information on patients with ccRCC, including age, sex, stage, tumor-node-metastasis stage, grade, survival status, and follow-up time. The ICGC (International Cancer Genome Consortium) database includes 45 normal tissues adjacent to primary tumors and 91 primary tumor solid tissues.

### 2.2. Identification of CRLRs

Cuproptosis-related genes (CRGs) were selected according to previously published methods [3]. Our study identified ten CRGs—namely, FDX1, LIAS, DLD, LIPT1, DLAT, PDHA1, PDHB, MTF1, GLS, and CDKN2A. Based on the CRGs and lncRNA expression profiles, Spearman correlation coefficients were calculated to identify the CRLRs (|R| > 0.5; *p* < 0.05). Utilizing R and the packages “cluster profile”, “ggplot2”, and “enrichplot”, we analyzed the biological significance of differentially expressed CRLRs using GO and KEGG data.

### 2.3. Construction of the CRLR Prognostic Model by a Machine Learning Algorithm

LASSO is a machine learning algorithm based on regression. In this method, a regularization function is introduced to punish over-fitting on the basis of logistic regression, and the regression coefficient is compressed such that unnecessary or insignificant covariates can be automatically removed and refined model variables can be obtained [10,13,14].

Using the R package glmnet, the LASSO Cox regression technique demonstrated that CRLRs were distinctly associated with the overall survival (OS) of patients with ccRCC. Combining univariate and multivariate Cox regression analyses, CRLRs were associated with significant increases. The equation of risk score = (β1 × CRLR-1) + (β2 × CRLR-2) + … + (βn × CRLRs-n). By combining the CRLR prognostic signature with independent TCGA-KIRC factors, we developed a hybrid nomogram. Patients with ccRCC were divided into risk groups according to their CRLR expression levels. Based on the clinical variables and the CRLRs in the hybrid nomogram, ROC analyses were conducted to estimate the accuracy of the 1-, 3-, and 5-year OS.

### 2.4. Kaplan–Meier (K–M) Survival Analysis and Principal Component Analysis (PCA)

We examined patients with ccRCC based on CRLR signatures using K–M survival curves and PCA. In our analysis of the TIDE model for immunotherapy, we used half-maximal inhibitory concentrations (IC50) from the GDSC web pages to estimate the therapeutic response where the TIDE model could predict whether immunotherapy would succeed.

### 2.5. Cell Culture

Human renal cortex proximal tubule epithelial cells (HK-2) and RCC cell lines (786-O, SN12C, UO31, and Caki-1) were purchased from the Chinese Academy of Sciences and cultured in 1640 medium (Gibco, New York, NY, USA) with 10% fetal bovine serum (FBS; Ausgenes, Australia) at 37 °C and 95% humidity in a 5% CO_2_ cell incubator.

### 2.6. qPCR and RNA Isolation

Total RNA was isolated using RNAiso Plus Reagent (9108, Shanghai, China), according to the manufacturer’s instructions. Total RNA was used for cDNA synthesis using the PrimeScript RT Reagent Kit (RR037A, Shanghai, China). Gene expression was quantified using TB Green Premix Ex TaqII (RR820A, Shanghai, China). Sangon Biotech Co., Ltd. (Shanghai, China) synthesized all primers for qPCR (see Table 1). The PCR procedure was as follows: 40 cycles of 98 °C for 30 s, 98 °C for 5 s, and 60 °C for 5 s. β-Actin served as the internal reference for normalization. The expression levels were calculated using the 2^−ΔΔCt^ method.

### 2.7. Statistical Analysis

We used the R statistical package (version 4.0.2) for our analyses. A Wilcoxon test was conducted to compare the proportion of tumor-infiltrating immune cells. Chi-square tests were used to analyze the differences in the proportions of clinical characteristics. PCR data were analyzed with an independent sample *t*-test using the GraphPad Prism 8.0 software. Statistical significance was defined as *p* < 0.05.

## 3. Results

### 3.1. Identification of the Prognostic CRLR Signature in ccRCC

**Figure 1:**
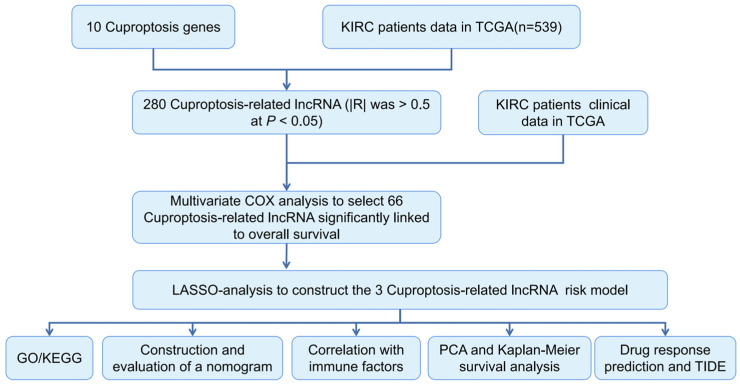
Research process flowchart.

**Figure 2:**
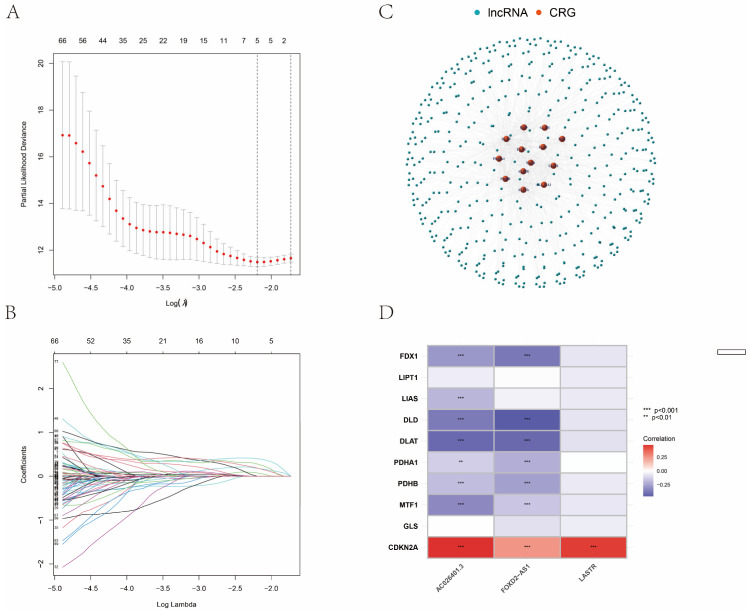
Identification of CRLRs. (**A**) The LASSO tuning parameters. (**B**) The CRLR LASSO coefficient profile. (**C**) Diagram of the coexpression network for cuproptosis genes and cuproptosis-related LncRNAs. (**D**) The heatmap for 10 cuproptosis genes with 3 cuproptosis-related LncRNAs. (** *p* < 0.01, *** *p* < 0.001).

**Figure 3:**
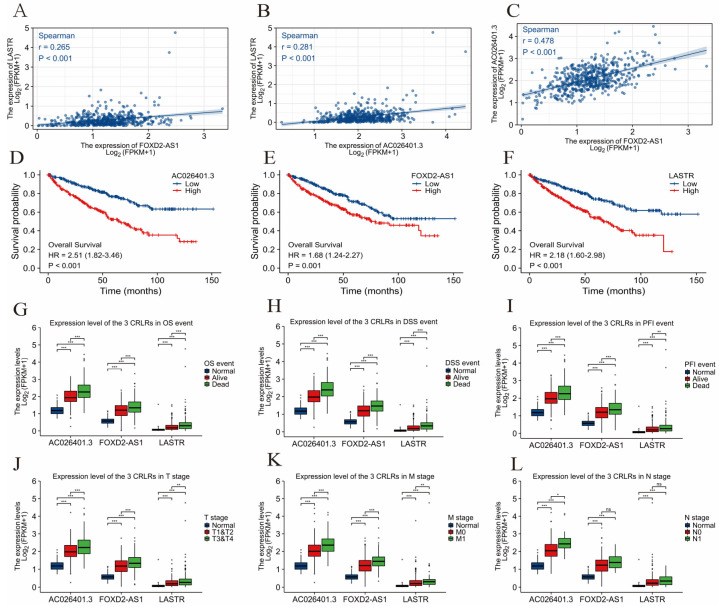
The clinical correlations analysis of the three CRLRs. (**A**) The molecular correlation of LASTR and FOXD2-AS1 in TCGA-KIRC. (**B**) The molecular correlation of LASTR and AC026401.3 in TCGA-KIRC. (**C**) The molecular correlation of FOXD2-AS1 and AC026401.3 in TCGA-KIRC. (**D**) K–M curves of AC026401.3 between the different expression level groups in TCGA-KIRC. (**E**) K–M curves of FOXD2-AS1 between the different expression level groups in TCGA-KIRC. (**F**) K–M curves of LASTR between the different expression level groups in TCGA-KIRC. (**G**) Expression level of three CRLRs in OS event. (**H**) Expression level of three CRLRs in DSS event. (**I**) Expression level of three CRLRs in PFI event. (**J**) Expression level of three CRLRs in T stage. (**K**) Expression level of three CRLRs in M stage. (**L**) Expression level of three CRLRs in N stage. (* *p* < 0.05, ** *p* < 0.01, *** *p* < 0.001).

**Figure 4:**
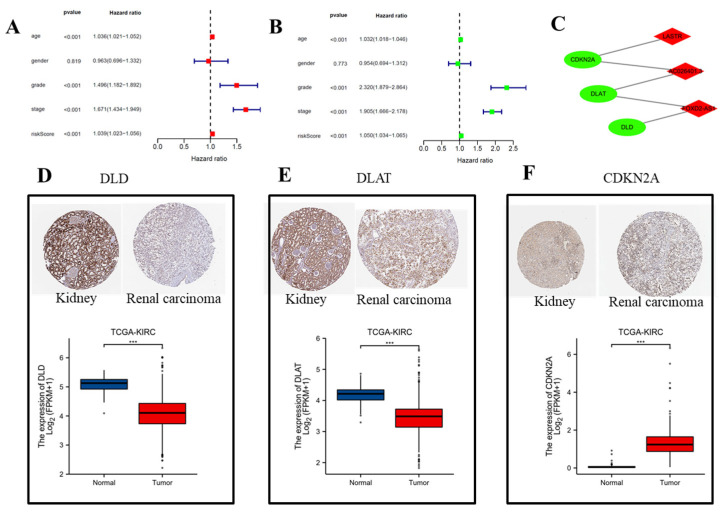
Independent prognostic analysis and validation of the effect of expression of real hub genes on transcriptional and translational level using TCGA database and The Human Protein Atlas database. (**A**) Univariate Cox with clinical variables and CRLRs. (**B**) Multivariate Cox with clinical variables and CRLRs. (**C**) Correlations between CRLRs and CRGs (*p* < 0.05). (**D**) Comparison of DLD in TCGA-KIRC tumor and normal kidney tissue. (**E**) Comparison of DLAT in TCGA-KIRC tumor and normal kidney tissue. (**F**) Comparison of CDKNA2 in TCGA-KIRC tumor and normal kidney tissue. (*** *p* < 0.001).

According to TCGA and previously published literature, 280 CRLR genes were identified (252 up-regulated and 28 down-regulated). Together with a multivariate Cox analysis, a univariate Cox analysis revealed 66 significant increases in CRLRs (Figure 1). The expression of three LncRNAs (AC026401.3, FOXD2-AS1, and LASTR) was found to be independent of prognosis in ccRCC by LASSO (Figure 2A,B). Figure 2C shows the network diagram for CRGs and CRLRs. Figure 2D shows the correlation heatmap of CRLRs and CRGs. Molecular correlation of the three CRLRs in TCGA-KIRC showed a significantly positive correlation (Figure 3A–C), whereas the KM prognostic curve (Figure 3D–F), overall survival event (Figure 3G), progression-free interval (Figure 3H), disease-specific survival (Figure 3I), and TMN stage (Figure 3J–L) presented significant differences. The multivariate Cox analyses revealed that CRLRs (hazard ratio (HR): 1.039, 95% confidence interval (CI): 1.023–1.056), age (HR: 1.036, CI: 1.021–1.052), grade (HR: 1.496, CI: 1.182–1.892), and stage (HR: 1.671, CI: 1.434–1.949) were prognostic factors for OS (Figure 4A). The univariate Cox analyses revealed that CRLRs (HR: 1.050, CI: 1.034–1.065), age (HR: 1.032, CI: 1.018–1.046), grade (HR: 2.32, CI: 1.879–2.864), and stage (HR: 1.905, CI: 1.666–2.178) were independent prognostic factors for OS (Figure 4B). Figure 4C shows the associations between CRLRs and CRGs. Based on the Oncomine database, we found that the expression of real hub genes was significantly elevated in renal carcinoma compared with normal tissues. Moreover, immunohistochemistry staining obtained from The Human Protein Atlas database also demonstrated the de-regulation of real hub gene expression (Figure 4D–I).

### 3.2. Construction of the Hybrid Nomogram and GO Analysis

**Figure 5:**
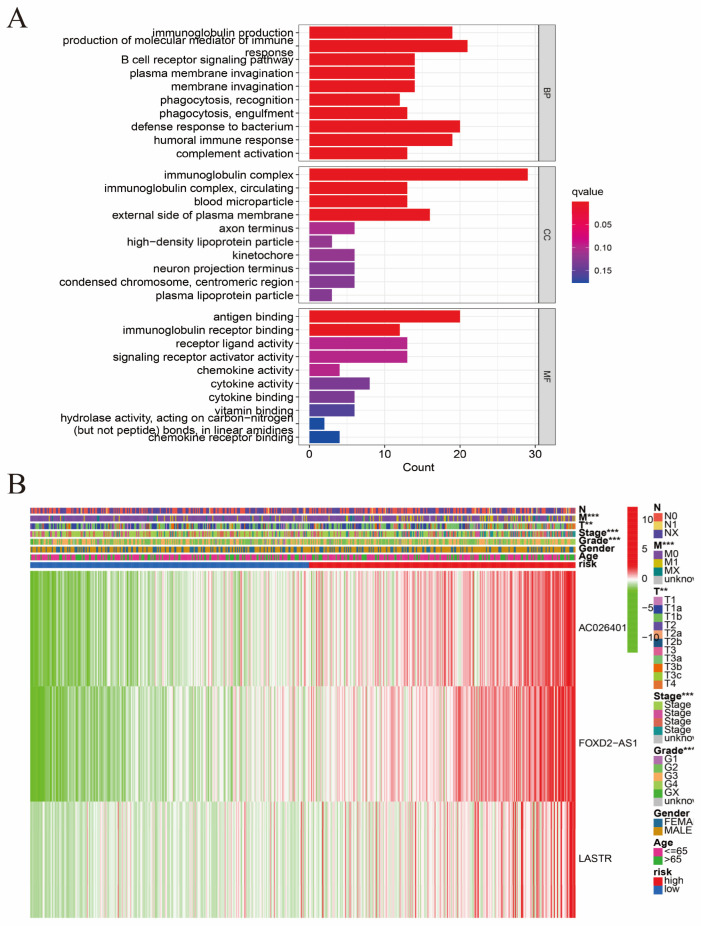
Enrichment analysis for CRLRs obtained from GO and correlation analysis. (**A**) GO enrichment analysis. (**B**) Heatmap of clinicopathological and biological characteristics of two different risk group subtypes of samples divided by the CRLR model. Differences in clinicopathologic features and expression levels of CRLRs between the two different risk groups. Red represents the high-risk group and blue represents the low-risk group. High lncRNA expression levels are shown in red and low lncRNA expression levels are shown in green. The three CRLRs showed a high expression trend in the high-risk group. CRLRs, cuproptosis-related lncRNAs. (** *p* < 0.01, *** *p* < 0.001).

**Figure 6:**
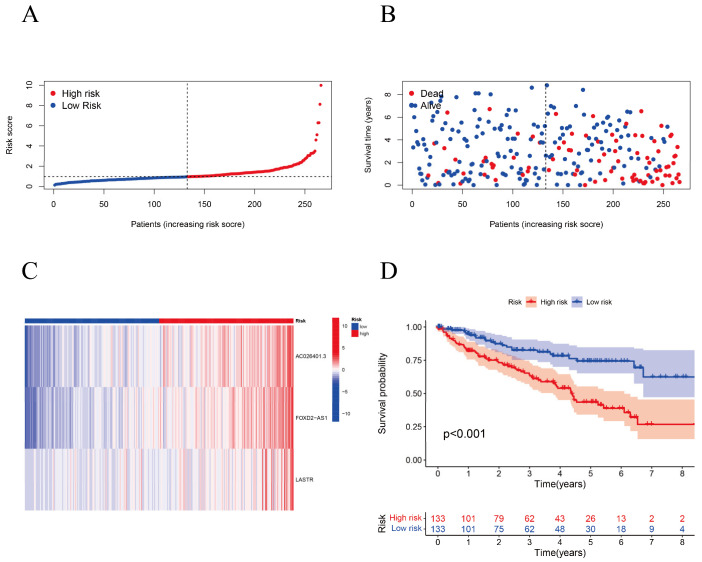
Development of a CRLR risk model in ccRCC. (**A**) The distribution of the risk grades between the low- and high-risk groups. (**B**) The survival statistics and survival times of the patients in the two risk groups. (**C**) The relative expression standards for the three CRLRs. (**D**) K–M survival curves of ccRCC in the low-risk group and the high-risk group (*p* < 0.001).

**Figure 7:**
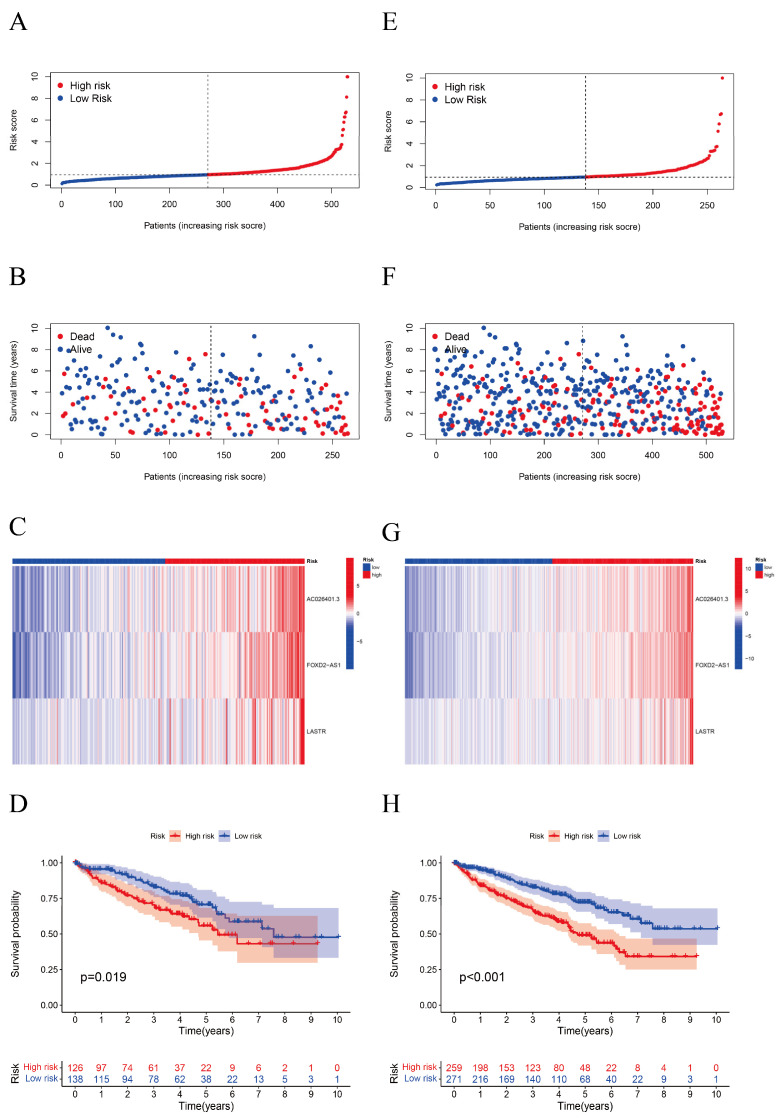
Validation of a CRLR risk model using the testing data set and the entire TCGA-KIRC data set. (**A**) Risk score distribution in the testing set. The red dots represent the high-risk group and the blue dots represent the low-risk group. (**B**) OS status for the testing set. The red dots represent dead patients and the blue dots represent living patients. (**C**) Heatmap for the testing set. (**D**) Kaplan–Meier curve for OS for the testing set. (**E**) Risk score distribution for the entire data set. (**F**) OS status for the entire TCGA-KIRC data set. (**G**) Heatmap for the entire TCGA-KIRC data set. (**H**) Kaplan–Meier curve for OS for the entire TCGA-KIRC data set. The red and blue lines represent high and low expressions, respectively. All *p* values are shown in Figure 7.

**Figure 8:**
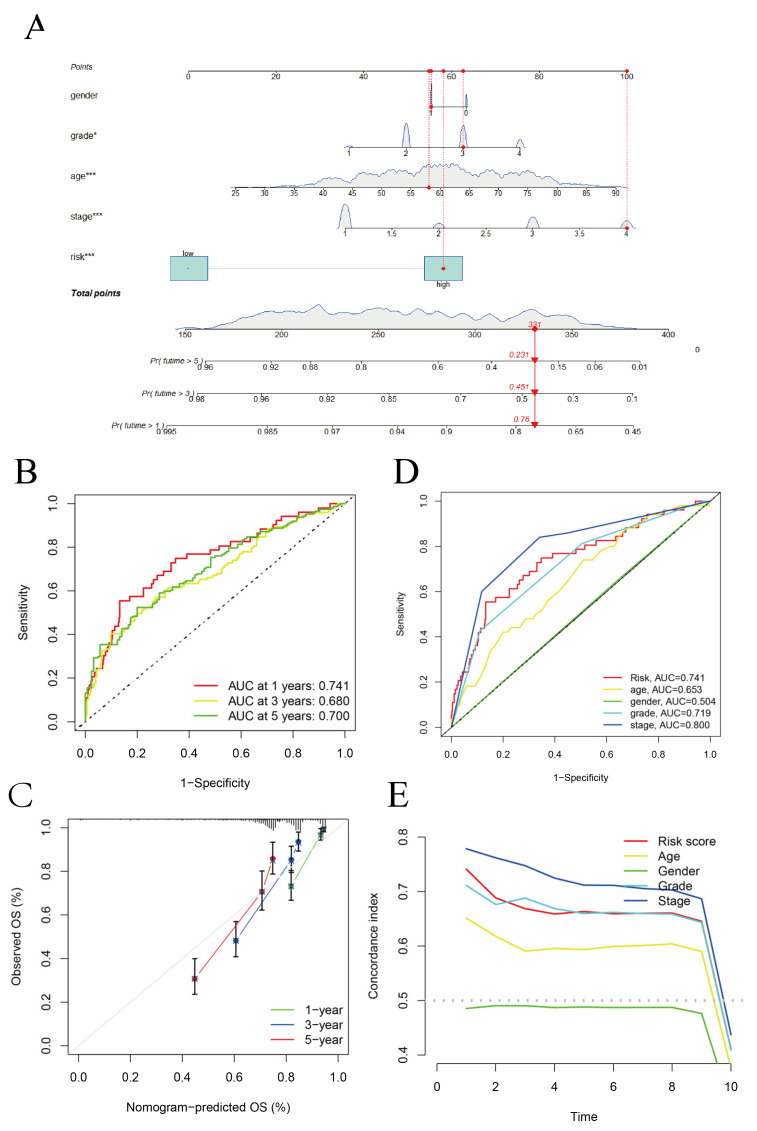
Nomogram, AUC, and DCA analysis. (**A**) Based on the selected CRLR prognostic signature and independent factors in ccRCC (* *p* < 0.05, *** *p* < 0.001). (**B**) The OS of AUC predictive for 1 year, 3 years, and 5 years. (**C**) Calibration plot of the CRLR nomogram. (**D**) The AUC of CRLRs and traditional clinical variables. (**E**) DCA plot of the CRLRs and traditional clinical variables.

A GO enrichment analysis revealed the involvement of many immune-related biological processes (Figure 5A). The novel CRLRs were involved in the production of immune-response molecular mediators, the defense response to bacteria, the humoral immune response, the immunoglobulin complex, the external side of plasma membrane antigen binding, and receptor–ligand activity. In Figure 5B, we present the correlations between CRLR features and clinical variables. Figure 6A illustrates the distribution of risk grades between the low- and high-risk groups. The survival statistics and survival times of the patients in the two risk groups are shown in Figure 6B. For each patient, Figure 6C shows the relative expression standards for the three CRLRs. Figure 6D shows that the low-risk group’s OS was greater than that of the high-risk group (*p* < 0.001). Using a uniform formula for every patient in the test set and for the entire data set, we calculated the risk scores for this established model in order to test its prognostic capabilities. Figure 7A–D illustrate the risk grades, survival times, survival status, and CRLR expression within the testing set and the entire KIRC sample (Figure 7E–H). Three CRLRs’ prognostic signatures and independent factors were combined to construct a hybrid nomogram for ccRCC (Figure 8A). The area under the curve (AUC) of OS was predictive for 1 year (0.741), 3 years (0.68), and 5 years (0.70) (see Figure 8B). Figure 8C shows the calibration plot of the nomogram. Additionally, the CRLR signature outperformed traditional clinical variables in predicting ccRCC patients (Figure 8D). As depicted in Figure 8E, the concordance index showed that the risk model performed better than the other clinical factors.

### 3.3. Survival Analysis and Principal Component Analysis

**Figure 9:**
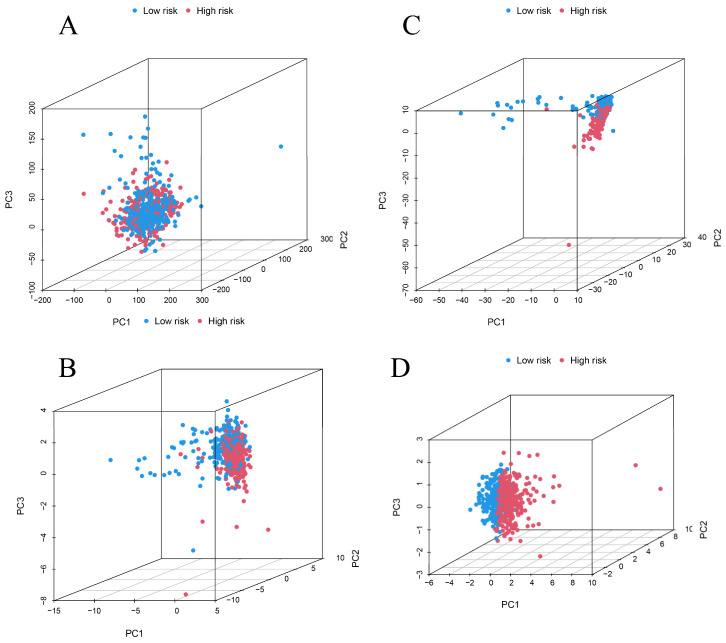
PCA analysis. (**A**) PCA of all genes. (**B**) PCA of 10 cuproptosis genes. (**C**) PCA of 280 CRLR genes. (**D**) PCA of three CRLRs.

**Figure 10:**
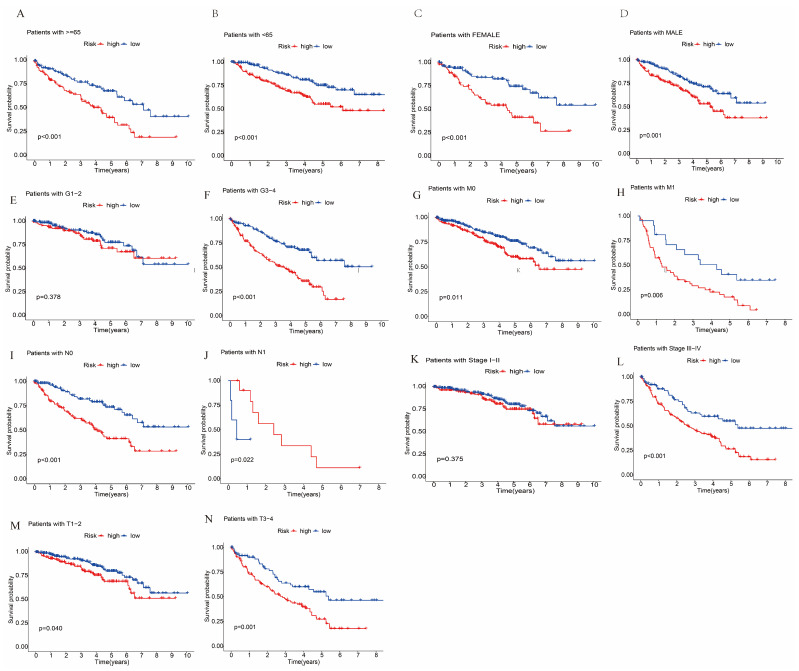
K–M curves of different clinical variables between the high-risk and low-risk groups of ccRCC patients in TCGA. (**A**) K–M curves for age ≥ 65 for the different risk groups of ccRCC patients. (**B**) K–M curves for age < 65 years for the different risk groups of ccRCC patients. (**C**) K–M curves for female for the different risk groups of ccRCC patients. (**D**) K–M curves for male for the different risk groups of ccRCC patients. (**E**) K–M curves for stages G1–2 for the different risk groups of ccRCC patients. (**F**) K–M curves for stages G3–4 for the different risk groups of ccRCC patients. (**G**) K–M curves for stage M0 for the different risk groups of ccRCC patients. (**H**) K–M curves for stage M1 for the different risk groups of ccRCC patients. (**I**) K–M curves for stage N0 for the different risk groups of ccRCC patients. (**J**) K–M curves for stage N1 for the different risk groups of ccRCC patients. (**K**) K–M curves for stages I–II for the different risk groups of ccRCC patients. (**L**) K–M curves for stages III–IV for the different risk groups of ccRCC patients. (**M**) K–M curves for stages T1–2 for the different risk groups of ccRCC patients. (**N**) K–M curves for stages T3–4 for the different risk groups of ccRCC patients.

Figure 9 provides diagrams showing the gene expression profiles of 2 types of ccRCC patients (Figure 9A), 10 cuproptosis genes (Figure 9B), 280 CRLR genes (Figure 9C), and 3 lncRNA risk models (Figure 9D). The three CRLR models can be seen to be excellent tools for distinguishing high- from low-risk patients with ccRCC. As shown in Figure 10A–N, by stratifying patients by tumor-node metastasis stage, sex, age, and grade, the K–M curve indicated that low-risk patients had a significantly better OS than high-risk patients (*p* < 0.001).

### 3.4. TIDE Algorithm and IC50 for Assessing Therapeutic Response

**Figure 11:**
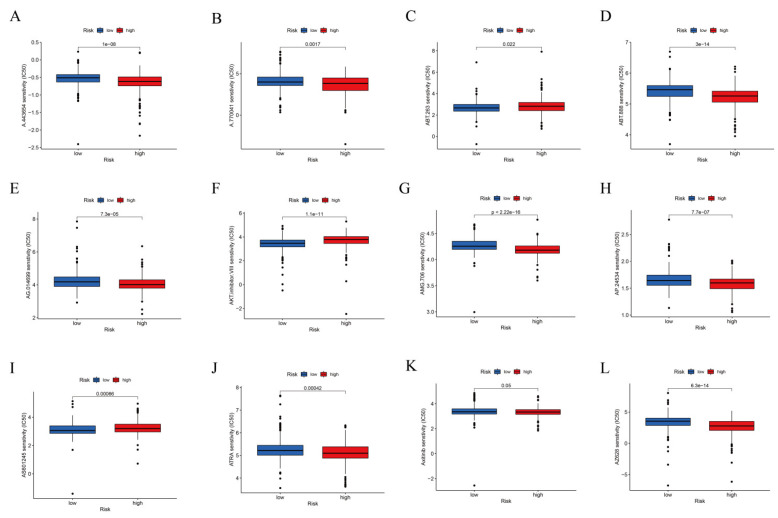
IC50 and therapeutic response analysis. The low-risk group is shown in blue on the abscissa, and the high-risk group is shown in red. The IC50 value of drug target sensitivity is shown on the ordinate. (**A**) A.443654. (**B**) A.770041. (**C**) ABT.263. (**D**) ABT.888. (**E**) AG.014699. (**F**) AKT inhibitor VIII. (**G**) AMG.706. (**H**) AP.24534. (**I**) AS601245. (**J**) ATRA. (**K**) Axitinib. (**L**) AZ628. The detailed *p* values are shown in Figure 11.

A prophetic algorithm was used to assess potential drug targeting for ccRCC using the three CRLR models. Low-risk participants were more sensitive to the 12 compounds, with significant differences based on the estimated IC50 values. The 12 different compounds in Figure 11A–L can thus be used to further analyze patients with ccRCC. The low-risk group was more sensitive to nine target drugs (A.770041, AG.014699, AMG.706, ATRA, AP.24534, axitinib, AZ628, ABT.888, and A.443654), based on the estimated IC50, and the high-risk group was more sensitive to ABT.263 and AKT inhibitors VIII and AS601245.

**Figure 12:**
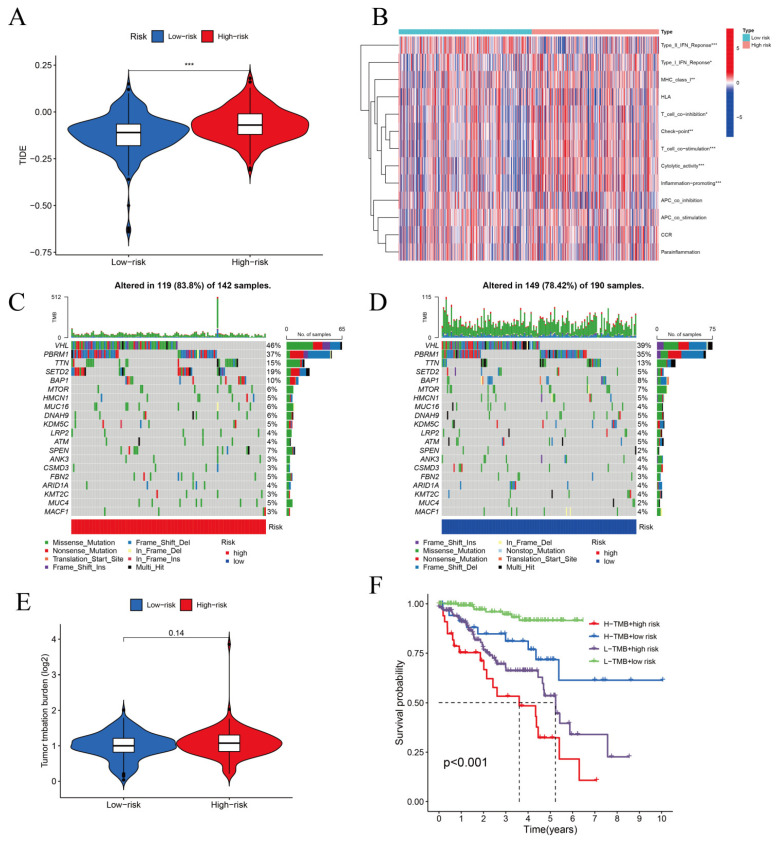
TIDE, Immunotherapy, Mutations, TMB, and Kaplan–Meier survival analysis. (**A**) TIDE analysis for the high- and low-risk groups (*** *p* < 0.001). (**B**) Immune indicators for the high- and low-risk groups. (**C**) Top 20 driver genes for the high-risk group. (**D**) Top 20 driver genes for the low-risk group. (**E**) TMB analysis for the two risk groups. (**F**) Kaplan–Meier survival with TMB status and risk level.

In TCGA-KIRC, we estimated the immunotherapy response based on the CRLR model. High-risk patients responded better to immunotherapy than low-risk patients, indicating that the cuproptosis-based classifier index may be useful in predicting tumor immune dysfunction and exclusion (Figure 12A). As shown in Figure 12B, the high- and low-risk groups expressed immune indicators differently. Maftools was used to analyze and summarize the mutation data; mutations were categorized according to the variant effect predictor. Figure 12C,D show the top 20 driver genes that were altered most frequently between the high- and low-risk sub-groups, and TMB scores were calculated based on the TGCA somatic mutation data. There was no difference between the high- and low-risk groups, indicating that the CRLR classifier index did not correlate well with TMB (Figure 12E). High TMB in KIRC was associated with a lower survival rate in high-risk patients (Figure 12F). Therefore, the results show that the CRLR model may be more predictive than the TMB status.

### 3.5. Validation of CRLRs by qPCR and ICGC Database

**Figure 13:**
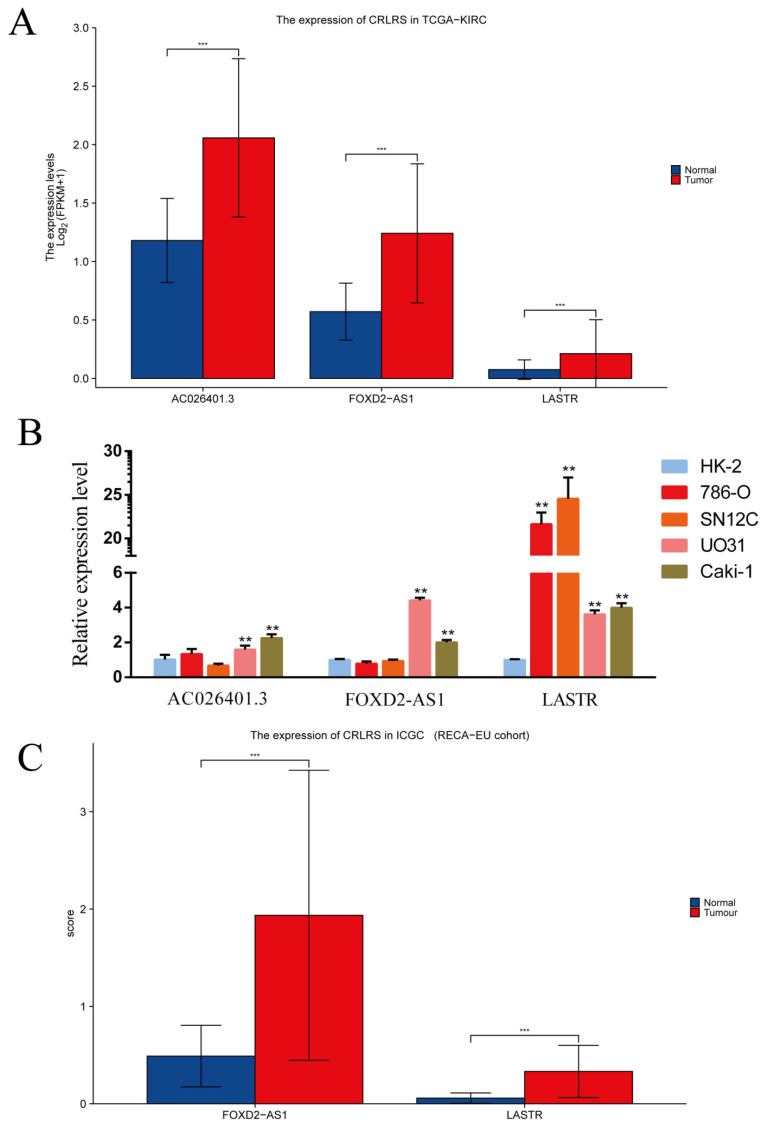
Validation of CRLRs in renal cancer. (**A**) Expression of CRLRs in TCGA-KIRC. (**B**) qPCR validation of CRLR expression levels in normal and renal cancer cells and expression levels of three CRLRs in HK-2, UO31, 786-O, SN12C, and Caki-1 cells (** *p* < 0.01, *** *p* < 0.001). (**C**) Expression of CRLRs in the ICGC (RECA-EU) cohort.

We evaluated the expression levels of the three core CRLRs using qPCR. The results showed that, compared with that in the proximal tubular cell line HK-2, the AC026401.3 level was significantly higher in the ccRCC cell lines (*p* < 0.01) UO31 and Caki-1. However, there was no significant change in the 786-O and SN12C cells (*p* > 0.05). The FOXD2-AS1 level was significantly higher in the UO31 and Caki-1-cell lines (*p* < 0.01), but again, there was no significant change in the 786-O and SN12C cells (*p* > 0.05). In particular, the LASTR level was very high in all four ccRCC cell lines (*p* < 0.01), ranging from 3.6 to 24.5 times that in the control cells. We further evaluated the expression levels of the CRLRs using the ICGC (RECA-EU) cohort. The expression of FOXD2-AS1 and LASTR was consistent with the PCR and TCGA-KIRC results (Figure 13).

## 4. Discussion

With the rapid popularization of artificial intelligence (AI) technology, it has shown strong application prospects in medicine. As such, artificial intelligence is gradually leading a revolution in the medical field. For example, in recent years, artificial intelligence has also garnered considerable interest in the field of tumor data processing [15,16]. The latest developments in biological sequencing technologies provide opportunities for a large amount of data mining in cancer research. However, due to the large amount of clinical data, it is difficult to carry out tumor research using traditional statistical analysis methods. How to use these clinical data to better carry out tumor research is a current focus of scientific research. Artificial intelligence technology based on machine learning technology allows for the extraction of data features from massive quantities of data and more accurate construction of risk stratification models for tumor patients, thereby assisting physicians in clinical decision making. Therefore, with the help of machine learning technology, the collection and mining of the available tumor data to find internal connections and rules has brought unprecedented opportunities to tumor research and diagnosis [9,10,11,12,17,18].

Machine learning is an interdisciplinary subject, especially in the statistical analysis of clinical medicine, which has become a research hotspot. Researchers think LASSO is a branch of regression analysis in machine learning. Of course, the application of the LASSO algorithm would be more appropriately described as a feature screening algorithm in machine learning. After screening variables, we retained the three most meaningful LncRNAs to build a prediction model. The expression of the three lncRNAs related to cuproptosis was verified in the TCGA, ICGC, and several tumor cell lines, consistent with the model prediction. We confirmed the expression of the three LncRNA indicators in tumor cells in the model, which was compatible with the expression in the database. According to the ROC curves for ccRCC in the TCGA database for 1 year, 3 years, and 5 years, the model’s prediction accuracy was 0.74, 0.68, and 0.70, respectively. In addition, compared with the traditional tumor risk assessment indicators, such as the ROC curve for TNM stage and age prediction, the risk model in this study had the best predictive ability (AUC = 0.741).

FOXD2-AS1 is a cancer-related gene [19] which is aberrantly expressed in various cancers and has been linked to cancer progression [20], targeting P53 [21], Akt/E2F1 [22], miR-25-3p/Sema4C [23], the microRNA-98-5p/CPEB4 axis [24], and PI3K/Akt [25]. Moreover, FOXD2-AS1 over-expression has been shown to lead to antitumor drug resistance in various cancers, including esophageal squamous-cell carcinoma [26] and hepatocellular carcinoma [27]. Thus, FOXD2-AS1 is an oncogene involved in a wide range of biological effects in cancer. We found that FOXD2-AS1 was present at high levels in ccRCC cells, indicating that FOXD2-AS1 is a potential target of cuproptosis in ccRCC, and that its mechanism is associated with signal alteration in the tumor microenvironment. Furthermore, FOXD2-AS1 reinforces the progression of rheumatoid arthritis by regulating the miR-331-3p/PIAS3 pathway [28]. In oral squamous-cell carcinoma, FOXD2-AS1 is negatively associated with B cells, DCs, iDCs, and mast cells [29].

LASTR is an lncRNA associated with the regulation of splicing by SART3. It plays an essential role in regulating plant metabolisms. However, research has demonstrated that LASTR modulates the activity of the U4/U6 recycling factor SART3 to boost cancer fitness [30], and also modulates the activity of the miR-137/TGFA/PI3K/AKT axis to accelerate lung cancer progression [31]. However, no study has reported a direct correlation between LASTR and immunity. Interestingly, LASTR has also been used as a ferroptosis-related marker in stomach adenocarcinoma [32]. Therefore, there are some connections between cuproptosis and ferroptosis, and LASTR could be involved in the cross-talk between them, thus playing an essential role in the immunological therapy of cuproptosis and ferroptosis.

According to previous studies, AC026401.3 is a glycolysis-based lncRNA predictor for prognosis in kidney [33] and liver cancer [34]. AC026401.3 regulates the immune response, as tumor-cell-induced glucose deprivation inhibits T-cell glycolysis and immunogenic functions [35]. Although we confirmed that AC026401.3 expression was elevated in ccRCC cell lines, the function of AC026401.3 is far from known at present.

Using the three CRLR models, potential drug targets for ccRCC were determined using the TIDE algorithm, and it was shown that low-risk patients were more sensitive to the 12 target drugs based on IC50 estimates. However, the results from the model of the tumor immune microenvironment and immunotherapy response showed that immunotherapy was more likely to work for high-risk patients than low-risk patients, suggesting that the cuproptosis-based classifier index could be used to predict immune response. Using somatic mutation data from TGCA, we calculated TMB scores. The low-risk group did not surpass the high-risk group, suggesting poor correlation with the CRLR classifier index. In ccRCC, a high TMB score with high risk was associated with a worse outcome and could be used as a prognostic marker. Thus, these findings demonstrate that the CRLR model has a higher prognostic value than TMB status. Therefore, AC026401.3, FOXD2−AS1, and LASTR might be useful indicators for investigating different drug treatments with different TMN stages and mutation load burdens, providing a foundation for the precise treatment of ccRCC. It should be emphasized that the specific functions and mechanisms of these three molecules (i.e., FOXD2−AS1, AC026401.3, and LASTR) in renal cancer still require further experimental studies.

According to the three CRLR models, the low-risk group was more sensitive to nine target drugs (A.770041, AG.014699, AMG.706, ATRA, AP.24534, axitinib, AZ628, ABT.888, and A.443654) based on the estimated IC50, whereas the high-risk group was more sensitive to ABT.263 and AKT inhibitors VIII and AS601245. The results showed that ccRCC patients with different expression levels of the three CRLRs presented different sensitivities to different target drugs, reflecting individual differences and tumor heterogeneity in kidney cancer patients. Considering the heterogeneity in immune response, it is suggested that targeted therapy combined with immunotherapy can provide precision treatment in ccRCC patients, based on different levels of the LncRNAs FOXD2−AS1 and AC026401.3. Thus, this study can provide a reference paradigm for various tumors, facilitating the mining of cuproptosis-related LncRNAs based on TCGA data. We must point out that although AC026401.3, FOXD2−AS1, and LASTR were confirmed to be highly expressed in ccRCC cell lines, most of the results in this study were only based on our CRLR models. Hence, more experimental studies are needed, especially regarding how the three CRLRs regulate cuproptosis-related genes. It is important to determine how the signal transduction pathways are regulated by AC026401.3, FOXD2−AS1, and LASTR, as well as the relationships between CRLRs and apoptosis, necroptosis, pyroptosis, ferroptosis, and autophagy. In addition, we investigated whether there were synergistic effects between each of the 12 targeted drugs and immunotherapy drugs. Moreover, the roles of these three CRLRs in the diagnosis, treatment, and prognosis of ccRCC need to be studied in multiple clinical centers and with a large number of samples.

There are also some limitations to this study. First of all, all the analyses are based on data from public databases, and all the samples used in this study were retrospectively obtained. Therefore, the inherent bias in case selection may have affected the outcome. It is necessary to conduct large-scale prospective studies as well as additional experiments in vitro and in vivo in order to confirm our findings. Moreover, many datasets did not include key clinical variables such as surgery, radiotherapy, and neoadjuvant chemotherapy, which may have affected accuracy.

## 5. Conclusions

We identified cuproptosis-related LncRNAs using a machine learning approach and investigated their potential value in ccRCC immunotherapy. We verified 280 CRLRs and identified 66 significant increments associated with CRLRs, according to multiple analysis models. The enrichment results demonstrated that CRLRs are involved in the production of immune responses molecular mediators, the defense responses to bacteria, the humoral immune response, the immunoglobulin complex, and receptor–ligand activity. Subsequently, we revealed that three different LncRNAs (FOXD2−AS1, AC026401.3, and LASTR) are prognostic predictors in TCGA-KIRC. The three molecules were further validated via qPCR and were found to be over-expressed in ccRCC cell lines. The ICGC includes 89 projects in 17 administrative regions of Asia, Australia, Europe, North America, and South America, including 25,000 cancer genomes. FOXD2−AS1 and LASTR were validated in the ICGC (RECA-EU) cohort. The results of the above two verification studies were consistent. In summary, we revealed that the core cuproptosis-related LncRNAs, FOXD2−AS1, AC026401.3, and LASTR, have potential prognostic value and can be used in a potential immunotherapeutic strategy to improve ccRCC outcomes.

## Figures and Tables

**Table 1:** Primers for qPCR.

Gene	Forward Primer	Reverse Primer
*LASTR*	3′-GCAAGAGAGAAGACAGTGGGTGAAG-5′	3′-CCAGTGAAGGGCTGAAGGGTTTAG-5′
*FOXD2−AS1*	3′-TGGGTTGAGGGTCTGTGACTGTAG-5′	3′-GCTGCCGCTGGAGTATTCTTGG-5′
*AC026401.3*	3′-AGTGGGAAATCTGACCTCTTTTGGC-5′	3′-TCCTGTTCTTAGTGGCTGCATTACC-5′
*β* *-Actin*	3′-CGGGAAATCGTGCGTGAC-5′	3′-CAGGAAGGAAGGCTGGAAG-5′

## Data Availability

The TIDE (http://tide.dfci.harvard.edu/) (version: accessed on 18 September 2020) and TCGA databases (https://portal.gdc.cancer.gov/repository) (v32.0 accessed on 29 March 2022) contain the datasets used to support this study.

## Data Availability Statement

The authors confirm that the data supporting the findings and conclusions of this study are available in the article.

## Abbreviations

lncRNAs	Long noncoding RNA
ICGC	International Cancer Genome Consortium
TCGA	The Cancer Genome Atlas
LASSO	Least absolute shrinkage and selection operator
ML	Machine learning
IC50	Half maximal inhibitory concentration
ccRCC	Clear cell renal cell carcinoma
CRLR	Cuproptosis-related long noncoding RNA
TIDE	Tumor immune dysfunction and exclusion
TMB	Tumor mutational burden
CRGs	Cuproptosis-related genes
PCA	Principal component analysis
OS	Overall survival
AUC	Area under the curve
